# OA12.02. CAM utilization among underserved patients in a safety-net hospital

**DOI:** 10.1186/1472-6882-12-S1-O46

**Published:** 2012-06-12

**Authors:** P Gardiner

**Affiliations:** 1 Boston Medical Center, Boston, USA

## Purpose

Little is known about the relationship between complementary and alternative medicine (CAM) usage and factors associated with underserved patients, such as health literacy, homelessness, alcohol and drug use, and high depression status.

## Methods

This is a secondary database analysis of the Project RED dataset (N=623) identifying the sociodemographic characteristics as well as underserved patient factors associated with CAM use. We used descriptive statistics, bivariate, and multivariate logistic regression. We used multivariate logistic regression models for each outcome (any CAM use, CAM provider delivered therapies, and relaxation practices), testing the association with independent variables associated with underserved patients.

## Results

In this underserved population (N=623), CAM use was reported by 51% of the non-Hispanic blacks, 40% of Hispanics, and 53% of the non-Hispanic whites. Twenty-seven percent used any relaxation techniques, and 28% used any practitioner delivered therapy. In this sample, individuals with a higher health literacy (> 9^th^ grade) were approximately three times [odds ratio 2.97, confidence interval (1.82, 4.86)] more likely to use relaxation techniques compared to those with a lower health literacy. Those who used illegal drugs were more likely to use any CAM [odds ratio = 1.81 (1.14-2.88)] and relaxation techniques [odds ratio = 2.61 (1.56-4.38)]. Those with mild depression were three times [odds ratio = 2.86 (1.19, 6.83)] more likely to use only relaxation techniques compared to those with no depression.

## Conclusion

It is important for providers to recognize that underserved patients utilized CAM and more research is needed to understand the patterns of CAM use in relation to access to care.

